# Long-term exposure to PM_2.5_ and cardiorespiratory mortality: an ecological small-area study in five cities in Colombia

**DOI:** 10.1590/0102-311XEN071024

**Published:** 2025-04-25

**Authors:** Diana Marín, Víctor Herrera, Juan Gabriel Piñeros-Jiménez, Oscar Alberto Rojas-Sánchez, Sonia C. Mangones, Yurley Rojas, Jhon Cáceres, Dayana M. Agudelo-Castañeda, Néstor Y. Rojas, Luis Carlos Belalcazar-Ceron, Jonathan Ochoa-Villegas, María Leonor Montes-Mejía, Veronica Maria Lopera-Velasquez, Sanit María Castillo-Navarro, Alexander Torres-Prieto, Jill Baumgartner, Laura A. Rodríguez-Villamizar

**Affiliations:** 1 Facultad de Medicina, Universidad Pontificia Bolivariana, Medellín, Colombia.; 2 Universidad Industrial de Santander, Bucaramanga, Colombia.; 3 Facultad Nacional de Salud Pública, Universidad de Antioquia, Medellín, Colombia.; 4 Instituto Nacional de Salud, Bogotá, Colombia.; 5 Universidad Nacional de Colombia, Bogotá, Colombia.; 6 Universidad del Norte, Barranquilla, Colombia.; 7 Universidad de San Buenaventura Medellín, Bello, Colombia.; 8 Secretaría de Salud Pública, Alcaldía de Santiago de Cali, Cali, Colombia.; 9 Secretaría de Salud de Medellín, Medellín, Colombia.; 10 Secretaría de Salud, Alcaldía de Barranquilla, Barranquilla, Colombia.; 11 Gobernación de Santander, Bucaramanga, Colombia.; 12 McGill University, Montreal, Canada.

**Keywords:** Mortality, Particulate Matter, Long-term Effects, Land Use, Regression Analysis, Mortalidad, Material Particulado, Efectos a Largo Plazo, Usos del Suelo, Análisis de Regresión, Mortalidade, Material Particulado, Efeitos a Longo Prazo, Usos do Solo, Análise de Regressão

## Abstract

Long-term exposure to the fine particulate matter (PM_2.5_) is a risk factor for cardiorespiratory mortality. However, little is known about its distribution and health impact in large cities in low-middle-income countries where population exposure has increased during the last decades. This ecological study evaluated the association between PM_2.5_ concentration and adult cardiorespiratory mortality at the intraurban census sector (CS) level of Colombia’s five most populated cities (2015-2019). We estimated incidence rate ratios (IRR; per 5µg/m^3^) by fitting negative binomial regressions to smoothed Bayesian mortality rates (BMR) on PM_2.5_ predicted from land use regression (LUR) models, adjusting for CS demographic structure, multidimensional poverty index, and spatial autocorrelation. CS median PM_2.5_ ranged from 8.1µg/m^3^ in Bucaramanga to 18.7µg/m^3^ in Medellín, whereas Bogotá had the highest variability (IQR = 29.5µg/m^3^) and cardiorespiratory mortality (BMR = 2,560 per 100,000). Long-term exposure to PM_2.5_ increased cardiorespiratory mortality in Bucaramanga (IRR = 1.15; 95%CI: 1.02; 1.31), without evidence of spatial clustering, and cardiovascular (IRR = 1.06; 95%CI: 1.01; 1.12) and respiratory (IRR = 1.07; 95%CI: 1.02; 1.13) mortality in Medellín. Cardiorespiratory mortality spatially clustered in some Colombian cities and was associated with long-term exposure to PM_2.5_ in urban areas where the LUR models had the highest predictive accuracy. These findings highlight the need to incorporate high-quality, high-resolution exposure assessments to better understand the health impact of air pollution and inform public health interventions in urban environments.

## Resumen

La exposición prolongada a material particulado fino (MP_2,5_) es un factor de riesgo para la mortalidad cardiorrespiratoria. Sin embargo, se sabe poco sobre la distribución de MP_2,5_ y su impacto sobre la salud de la población que vive en las grandes ciudades de los países de ingresos medianos-bajos (donde la exposición ha aumentado en las últimas décadas). Este estudio ecológico evaluó la asociación entre la concentración de MP_2,5_ y la mortalidad cardiorrespiratoria en adultos de los sectores censales (SC) intraurbanos de las cinco ciudades más pobladas de Colombia (2015-2019). Estimamos la razón de las tasas de incidencia (RTI; por 5μg/m^3^), con ajustes de las regresiones binomiales negativas a las tasas de mortalidad bayesiana (TMB) suavizadas para MP_2,5_ que se estimaron a partir de modelos de regresión del uso de la tierra (RUT), ajustando la estructura demográfica del SC, el índice de pobreza multidimensional y la autocorrelación espacial. La mediana de MP_2,5_ por SC varió de 8,1μg/m^3^ en Bucaramanga a 18,7μg/m^3^ en Medellín. Por otra parte, Bogotá concentró la mayor variabilidad (IIQ = 29,5μg/m^3^) y mortalidad cardiorrespiratoria (TMB = 2.560 por 100.000). La exposición prolongada a MP_2,5_ aumentó la mortalidad cardiorrespiratoria en Bucaramanga (RTI = 1,15; IC95%: 1,02; 1,31), la única sin evidencia de agrupamiento espacial, y la mortalidad cardiovascular (RTI = 1,06; IC95%: 1,01; 1,12) y respiratoria (RTI = 1,07; IC95%: 1,02; 1,13) en Medellín. La mortalidad cardiorrespiratoria se agrupó espacialmente en algunas ciudades colombianas y se asoció con la exposición prolongada a MP_2,5_ en áreas urbanas donde los modelos RUT presentaron una mayor precisión predictiva, lo que destaca la necesidad de incorporar evaluaciones de exposición de alta calidad y alta resolución para comprender mejor el impacto de la contaminación del aire en la salud de la población e informar las intervenciones de salud pública en entornos urbanos.

## Resumo

A exposição prolongada a material particulado fino (MP_2,5_) configura um fator de risco à mortalidade cardiorrespiratória. No entanto, pouco se sabe sobre a distribuição de MP_2,5_ e seu impacto na saúde em grandes cidades de países de renda média-baixa (em que a exposição da população aumentou nas últimas décadas). Este estudo ecológico avaliou a associação entre a concentração de MP_2,5_ e mortalidade cardiorrespiratória em adultos no nível do setor censitário (SC) intraurbano das cinco cidades mais populosas da Colômbia (2015-2019). Estimamos as razões das taxas de incidência (RTI; por 5μg/m^3^), ajustando regressões binomiais negativas às taxas de mortalidade bayesianas suavizadas (TMB) para MP_2,5_ que foram previstas a partir de modelos de regressão do uso da terra (RUT), ajustando-se à estrutura demográfica do SC, índice de pobreza multidimensional e autocorrelação espacial. A mediana de MP_2,5_ por SC variou de 8,1μg/m^3^ em Bucaramanga a 18,7μg/m^3^ em Medellín. No entanto, Bogotá apresentou a maior variabilidade (IIQ = 29,5μg/m^3^) e mortalidade cardiorrespiratória (TMB = 2.560 por 100.000). A exposição prolongada a MP_2,5_ aumentou a mortalidade cardiorrespiratória em Bucaramanga (RTI = 1,15; IC95%: 1,02; 1,31) - a única sem evidência de agrupamento espacial - e mortalidades cardiovascular (RTI = 1,06; IC95%: 1,01; 1,12) e respiratória (RTI = 1,07; IC95%: 1,02; 1,13) em Medellín. A mortalidade cardiorrespiratória agrupou-se espacialmente em algumas cidades colombianas e foi associada à exposição prolongada ao MP_2,5_ em áreas urbanas onde os modelos RUT tiveram a maior precisão preditiva, destacando a necessidade de incorporar-se avaliações de exposição de alta qualidade e alta resolução para entender melhor o impacto da poluição do ar na saúde e informar as intervenções de saúde pública em ambientes urbanos.

## Introduction

Air pollution is a global public health issue that accounted for 6.7 million deaths worldwide in 2019 ^1^. The leading causes of ambient air pollution include population growth, urbanization, and industrialization in the face of incomplete adherence of governmental regulations to stricter emission standards ^2^. Exposure to ambient air pollution, specifically fine particulate matter (PM_2.5_), and its impact on disease burden have exhibited a sustained and concerning rise over the last two decades, mainly in countries with low and low-middle sociodemographic indices ^1^. This impact is primarily explained by the well-known association between PM_2.5_ exposure and cardiovascular and chronic respiratory morbidity and mortality ^3^ ^,^ ^4^ ^,^ ^5^, an effect recently confirmed in North American ^6^ ^,^ ^7^ ^,^ ^8^ and European ^9^ ^,^ ^10^ populations with exposure levels below the current air quality guidelines ^11^.

Studies conducted in Latin America provide compelling evidence confirming the relation between short-term exposure to PM_2.5_ and cardiorespiratory outcomes ^12^ ^,^ ^13^ ^,^ ^14^ ^,^ ^15^ ^,^ ^16^; however, these studies share among their main limitations the estimation of exposure based on remote sensors or monitoring networks with low spatial resolution. Importantly, no study has evaluated the association between long-term exposure to ambient PM_2.5_ and cardiorespiratory mortality in Latin America. However, a large nested case-control study conducted in Mexico found a 2.8 times higher risk of incident coronary artery disease per 5µg/m^3^ increase of 5-year mean residential PM_2.5_ after adjusting for cardiovascular risk factors ^17^. Although promising and informative, considering the high risk of fatal cardiovascular outcomes in such patients, the results from this study require replication and extension to other health outcomes by other research in the region.

Moreover, from a public health perspective, formulating and implementing successful environmental policies to reduce cardiorespiratory mortality, in the long run, will benefit from predictive models sensitive to intraurban, small-scale PM_2.5_ exposure heterogeneity ^18^ and suitable for regular updating from secondary data sources. This study evaluated the association between long-term exposure to PM_2.5_ estimated at the intraurban census sector (CS) level, using land use regression (LUR) models, and mortality from chronic cardiorespiratory diseases in adults from Colombia’s five most populated cities.

## Methods

### Study design, population, and geographical units

**Figure 1:**
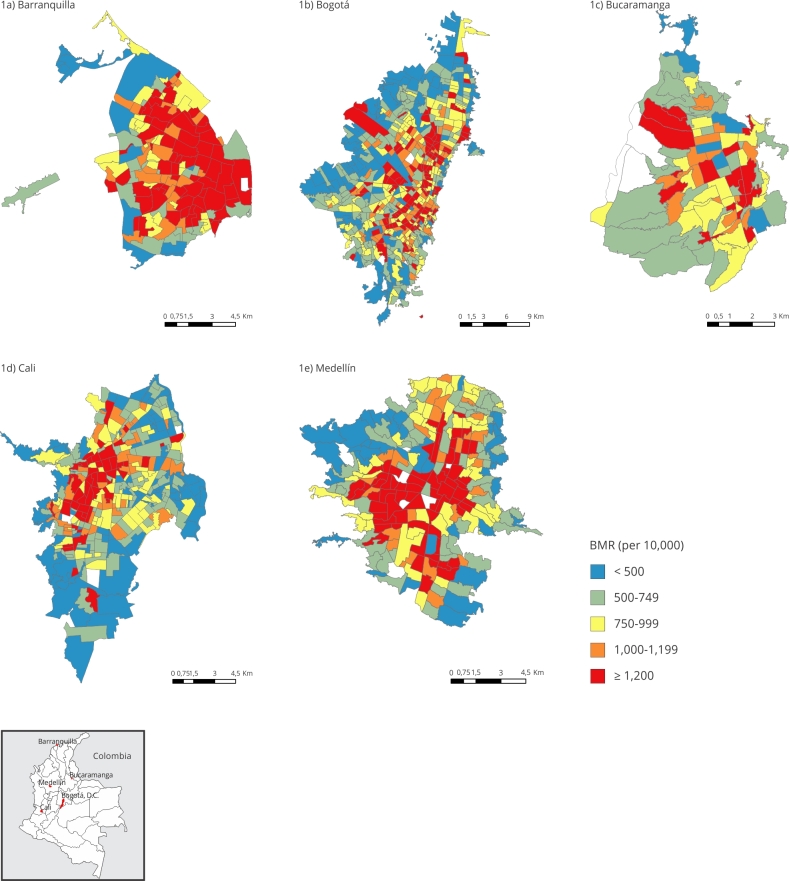
Bayesian cardiorespiratory mortality rates by city in Colombia, 2015-2019.

We conducted a cross-sectional ecological study examining mortality rates in Colombia’s five most populated cities. Colombia, situated in the northernmost part of South America, had an estimated population of 50 million people in 2019 ^19^. Urban areas accounted for 77.1% of the total population, with Bogotá, the capital district, being the most populous city, housing approximately 8 million residents. Other major cities included Medellín (population of 2.8 million), Cali (population of 2.5 million), Barranquilla (population of 750,000), and Bucaramanga (population of 600,000). Figure 1 shows the geographical locations of these five cities. The study population included adults aged 18 years and older residing in the urban areas of these cities who died between January 1, 2015, and December 31, 2019.

BMR: Bayesian mortality rates. Note: quintiles were split according to the index range values for all cities.

We employed CS as the geographical unit of analysis for this ecological study, which result from aggregating a number of census blocks established by the Colombian National Administrative Department of Statistics (DANE, acronym in Spanish) for census purposes. CS are the smallest available geographic units (median population = 5,831) containing population and census data that maintain a low risk of identifying individual cases (deaths). Importantly, CS exhibits variations in population size both within and between cities, making them heterogeneous units in terms of population. We accessed the DANE Geoportal public website to obtain the necessary cartographic information and maps, specifically utilizing the 2018 national census data ^20^. Spatial data were created using ArcGIS 10.8.1 (http://www.esri.com/software/arcgis/index.html), employing the custom azimuth equidistant projection and WGS 1984 datum, aligning with the geographic coordinate system used in Colombia.

### Mortality data

We obtained mortality data from the Colombian National Vital Statistics System, particularly from the Mortality Registry provided by the health authorities of the five cities which is centralized at the national level by DANE, codified in terms of causes of death, and then made available for all municipalities, including all deaths that occurred in the country by place of residency. The mortality registry used diagnostic codes from the 10th revision of the International Classification of Diseases (ICD-10). We included deaths of adults over 18 years old who died from the following selected cardiorespiratory diseases: cardiovascular diseases included angina pectoris (I20), acute myocardial infarction (I21-I24), conduction disorders (I44-I45), cardiac arrhythmias (I47-I49), heart failure (I50), and cerebrovascular diseases (I60-I69). The respiratory diseases included respiratory infections (J00-J06; J10-J18; J20-J22), asthma (J45-J46), and chronic obstructive pulmonary disease (J40-J44). These causes of death were chosen because they correspond to the leading causes of mortality related to the adverse effects of particulate matter ^21^. The counts of specific causes of death were obtained at the CS level between 2015 and 2019 based on the availability of a valid residence address in the death registry, a process that was internally performed by the local authorities, according to which 83%, 86%, 83%, 84%, and 87% of the addresses could be georeferenced in Barranquilla, Bogotá, Bucaramanga, Cali, and Medellín, respectively.

### PM_2.5_ long-term exposure

PM_2.5_ exposure levels were derived from LUR models available for the five cities for the year 2021. Details of the LUR models development have been described elsewhere ^22^. Briefly, measurement campaigns were simultaneously conducted across sampling sites in each city for two weeks during the dry and rainy seasons in 2021. There were 20 sampling sites for PM_2.5_ in all cities except for Bogotá, where 40 sampling sites were selected. The LUR models for each city were built using multivariable spatial regression models having as dependent variables the mean PM_2.5_ concentration measured at sampling points during both campaigns. Predictors of the models included land use data, population counts and density, road data (total length and distance), altitude, and meteorology. The estimated R^2^ of the models were 0.82 for Medellín, 0.77 for Bucaramanga, 0.73 for Barranquilla, 0.70 for Cali, and 0.44 for Bogotá ^22^. We estimated the CS-level long-term exposure to PM_2.5_ in 2021 by calculating the average of the 50x50 grid cells centroid points within each CS. Mean standard deviation of the estimated PM_2.5_ in the grid cells within CS was 1.11μg/m^3^ in Bucaramanga, 1.72μg/m^3^ in Bogotá, 1.95μg/m^3^ in Cali, 2.47μg/m^3^ in Medellín, and 2.86μg/m^3^ in Barranquilla.

### Demographic and socioeconomic data

Population data pertaining to individuals aged 18 and above, disaggregated by gender and age groups, as well as urban/rural residence status, were obtained at the CS level. These population estimates were derived from the comprehensive population estimations provided by the DANE census ^19^. Specifically, data for the year 2018 were chosen as this represents the most recent census year in Colombia, ensuring the availability of population counts at the CS level.

The socioeconomic context was assessed using the Colombian multidimensional poverty index (MPI) as the primary measure ^23^. The MPI, developed by DANE since 2011, serves as a composite index that captures poverty from a multi-deprivation perspective. It encompasses five dimensions, including schooling level, conditions of children and youth, employment, health, access to public utilities, and housing conditions. Notably, the health dimension incorporates aspects like health coverage and access while excluding mortality as one of its indicators. A proxy of the national MPI was constructed by DANE at both the municipal and CS levels using census data ^24^. For our study, we utilized the Colombian 2018 MPI, which falls within the study period and represents the most recent MPI measurement derived from national census data. This index, observed at the municipal and CS level, indicates the percentage of the population experiencing multidimensional poverty. Hence, a higher index value corresponds to a greater degree of socioeconomic deprivation.

### Statistical analysis

We computed cumulative crude mortality rates at the CS level as the quotient between death counts and population data for the year 2018, which served as the average population for the study period spanning from 2015 to 2019. However, due to the small size of some geographical areas, the crude rates could be unstable due to low counts. To address this, we employed a smoothed standardized mortality ratio (SMR) approach employing an empirical Bayesian method ^25^. First, we calculated the SMR for each CS using the formula: SMR = total number of deaths / expected total number of deaths. The expected number of total deaths, for all causes or specific causes of death, was determined as the product of the total population in the CS and the overall city mortality rate. Subsequently, we smoothed the SMRs to obtain smoothed SMRs, considering the unstable rates resulting from low death counts in some CS. This smoothing process was accomplished using empirical Bayes estimators within a Poisson random intercept regression model ^25^ ^,^ ^26^.

Employing the smoothed SMRs, we derived Bayesian mortality rates (BMRs) at the CS level. To explore the spatial patterns, we examined the spatial autocorrelation of the mortality outcome variables by employing Moran’s index. As described in a previous study, we generated the Moran eigenvector spatial filters (MESF) to identify spatial patterns in mortality distribution across the CS within cities ^22^. For processing geographical boundaries, we used a connectivity “queen” matrix for the CS, in which for each CS we included CS neighborhoods that shared boundaries on a single node (point) or a segment of border limits. We used the MESF to estimate the association between the BMRs and PM_2.5_ in a regression model while controlling for covariates and removing potential spatial autocorrelation present in the residuals ^27^ ^,^ ^28^. We described mortality counts and BMRs at the CS level using summary measures and visual representations through maps.

To estimate the overall functional association between air pollution exposure and mortality, we plotted the BMRs (overall cardiorespiratory, circulatory and respiratory causes) and air pollution exposure across CS by city. We assessed the SMRs distribution which showed strong overdispersion and did not fit a Poisson distribution. We then fit negative-binomial multivariable models including the BMRs as outcome variable and PM_2.5_ estimated long-term exposure centered at increments of 5μg/m^3^ as main exposure variable. We estimated incidence rate ratios (IRR) and their 95% confidence intervals (95%CI). The models were controlled for the effect of age and gender structure (dichotomous variable with cut-off value for 10% or more population of 60 years or above and dichotomous variable with cut-off value for 50% or more male population, respectively), quintiles of the MPI, and the spatial filter. We run separated models for mortality due to cardiorespiratory, circulatory, and respiratory diseases. All models were run using robust variance clustered by city to account for the natural aggregation of CS data.

We conducted a sensitivity analysis using progressive cumulative mortality rates from 2015-2019 (2015-2019, 2016-2019, 2017-2019, and 2018-2019) and its effect in the estimated IRR. These analyses were conducted to assess the consistency of findings with different periods of time including those closer to the year of exposure assessment. We used Stata, version 13 (https://www.stata.com), for calculating smoothed SMR and rates and fit multivariable models, eigenvector spatial filter tool software ^29^ for calculating Moran’s index and MESF, and ArcGIS to generate maps.

## Results

### Descriptive analysis of mortality and PM_2.5_

A total of 72,029 cardiorespiratory deaths involving adults were recorded during 2015-2019 in the five studied cities. The crude cumulative mortality rates vary across cities, ranging between 683.0 in Bogotá and 1,113.7 in Barranquilla. Deaths caused by diseases of the circulatory system were the most frequent, except in Cali where deaths were more frequently caused by respiratory than circulatory diseases. Table 1 shows the number of deaths, mortality rates, and BMRs at the city level and CS. Bogotá exhibited the highest BMRs from cardiorespiratory and respiratory causes (median BMR = 2,560.4 and 795.2 per 100,000, respectively) whereas Cali had the lowest BMR from circulatory diseases (median BMR = 260.9 per 100,000).

**Table 1:** Number of deaths, crude and Bayesian mortality rates (BMR) for all cardiorespiratory deaths, and specific causes of death at the city and census sector level for five cities in Colombia, 2015-2019.

Statistics	Barranquilla		Bogotá		Bucaramanga		Cali		Medellín	
	n	Rate (per 100,000)	n	Rate (per 100,000)	n	Rate (per 100,000)	n	Rate (per 100,000)	n	Rate (per 100,000)
Total cardiorespiratory deaths	8,477	1,113.7	35,900	683.0	3,287	871.3	9,341	706.5	15,024	869.3
Total circulatory deaths	5,699	748.7	22,842	434.5	2,063	546.8	3,405	257.5	8,627	499.2
Total respiratory deaths	2,778	365.0	13,058	248.4	1,224	324.4	5,936	449.0	6,397	370.1
Population over 18 years	761,183		5,256,596		377,262		1,322,156	706.5	1,728,348	
Census sectors included	145		607		85		328		227	
Statistics by census sector	Min-Max	Median (p25-p75)	Min-Max	Median (p25-p75)	Min-Max	Median (p25-p75)	Min-Max	Median (p25-p75)	Min-Max	Median (p25-p75)
Cardiorespiratory deaths	3-283	39 (19-85)	0-284	45 (23-87)	2-182	30 (16-50)	0-140	21 (10-39)	1-269	62 (31-94)
Circulatory deaths	1-183	28 (13-58)	0-174	29 (15-53)	1-109	21 (11-31)	0-140	8 (3-14)	0-151	34 (18-54)
Respiratory deaths	0-100	12 (6-27)	0-112	17 (8-31)	1-73	11 (6-18)	0-76	14 (6-26)	0-118	27 (10-42)
Cumulative cardiorespiratory mortality	125-13,660	1,128 (820-1,512)	0-21,053	795,7 (527.1-1,152.0)	384-6,664	864 (584.8-1,169.8)	0-86	714.9 (470.7-1,086.3)	18-18,919	909.9 (625-1,265.1)
Cumulative circulatory mortality rate	67-8,247	748.4 (532-971)	0-15,790	505.1 (321.2- 713.9)	168-3,991	504.0 (370.7-737.3)	0-6,902	256.4 (147.3-405.2)	0-7,282	512.7 (371.3-724.8)
Cumulative respiratory mortality rate	0-5,412	355.8 (222.6-515.9)	0-11,111	277.4 (180.7-433.3)	55-2,673	295.9 (219.4-468.5)	0-3,448	465.1 (273.0-689.4)	0-16,216	386.7 (241.9-563.9)
BMR - cardiorespiratory	247-11,410	1,122.3 (839.1-1,471.4)	71- 4,437	2,560.4 (1,777.1-3,479.4)	419-6,380	868.3 (648.3- 1,139.9)	0-4,755	727.2 (515.7-1,033.3)	43-8,463	908.8 (635.8-1,243.2)
BMR - circulatory	185-6,102	744.5 (574.6-932.6)	92-5,845	795.2 (547.1-1,108.8)	315-3,725	519.4 (418.7-717.8)	134-6,031	260.9 (193.2-364.0)	38-6,587	511.9 (379.4-707.9)
BMR - respiratory	93-3,522	356.8 (262.5-479.4)	60-3,012	505.9 (355.5-679.2)	130-2,413	316.2 (249.9-415.3)	142-9,691	465.8 (328.1-647.5)	28-4,146	385.5 (254.6-541.7)

Figure 1 show the geographic distribution of the BMRs from cardiorespiratory causes and Figures S1 and S2 (Supplementary Material; https://cadernos.ensp.fiocruz.br/static//arquivo/suppl-e00071024_9031.pdf) show the BMRs from circulatory and respiratory causes, respectively. CS with higher circulatory BMRs tended to concentrate towards the center and east in Barranquilla, at the expanded city center in Bucaramanga and Medellín, and scattered in clusters along the north-south axis in Bogotá and Cali. CS with higher respiratory BMRs usually aggregated to the east in Barranquilla and Bogotá, but slightly dispersed from north to south, clustered to the west in Cali, and dispersed in clusters along the north-south axis in Medellín. As for exposure, the median concentration of PM_2.5_ at the CS level ranged from 8.1μg/m^3^ in Bucaramanga to 18.7μg/m^3^ in Medellín (Table 2). Barranquilla had the lowest and highest PM_2.5_ concentrations at the CS level: 0.5 and 38.8μg/m^3^, respectively. Bogotá exhibited the highest exposure variability across CS (interquartile range - IQR = 8.9 to 38.4μg/m^3^).

**Table 2:** Census sector land use regression (LUR)-based predicted PM_2.5_ concentration for five cities in Colombia, 2021.

Statistics	Barranquilla	Bogotá	Bucaramanga	Cali	Medellín
Minimum	0.5	5.6	2.3	1.5	5.3
Maximum	38.8	71.9	25.3	30.3	33.0
Mean	14.0	10.9	8.0	9.4	18.7
Standard deviation	6.9	5.6	3.5	4.2	5.4
Median	13.6	9.7	8.1	10.4	18.7
Percentile 25th	9.7	8.9	6.2	6.0	15.1
Percentile 75th	17.0	38.4	9.0	11.6	22.1

### Regression models of mortality

Table 3 shows the main results from the multivariable analyses. We found positive statistically significant effects of PM_2.5_ on mortality in two cities: Bucaramanga and Medellín. Bucaramanga showed a significant increase of 15.5% per 5μg/m^3^ in cardiorespiratory mortality (95%CI: 2.0; 31.0) and 17.6% per 5μg/m^3^ in respiratory mortality (95%CI: 3.0; 34.0). We observed a significantly higher risk of circulatory and respiratory mortality in Medellín: 6.2% (95%CI: 1.0; 12.0) and 7% (95%CI: 2.0; 13.0), respectively. Regarding demographic indexes, there was a statistically significant ecological effect of the proportion of older individuals on mortality for all cities, but the effect of gender distribution was inconsistent. On the other hand, we found statistically significant relations between MPI on cardiorespiratory mortality in Cali (p-trend = 0.033), circulatory mortality in Barranquilla (p-trend = 0.028), and respiratory mortality in Bogotá (p-trend = 0.014) and Cali (p-trend = 0.008). Specifically, we observed higher IRR for circulatory and respiratory mortality in the MPI second and third quintiles compared with the first quintile (lower MPI; reference) in Bogotá and Medellín and a significantly higher IRR for the fifth compared with the first quintile in Cali. For Barranquilla, Bogotá, and Cali, but not for Bucaramanga and Medellín, the spatial filters had a statistically significant contribution in explaining the variability of the three outcomes.

**Table 3:** Association between PM_2.5_ and cardiorespiratory, circulatory, and respiratory Bayesian mortality rates for five cities in Colombia, 2015-2019.

Model estimations	Barranquilla		Bogotá		Bucaramanga		Cali		Medellín	
	IRR	95%CI	IRR	95%CI	IRR	95%CI	IRR	95%CI	IRR	95%CI
Cardiorespiratory deaths										
PM_2.5_	1.03	0.98; 1.08	0.97	0.94; 1.00	1.16	1.02; 1.31	0.99	0.93; 1.05	1.05	0.99; 1.11
% adults aged 60 or more years	1.43	1.22; 1.68	1.69	1.48; 1.93	1.52	1.13; 2.02	1.33	1.18; 1.50	2.09	1.72; 2.55
% male	0.40	0.27; 0.61	1.15	1.002; 1.31	1.28	0.56; 2.88	0.89	0.67; 1.17	2.78	1.59; 4.84
MPI (quintiles)										
Q1 (least deprived)	Reference		Reference		Reference		Reference		Reference	
Q2	1.08	0.89; 1.30	1.16	1.02; 1.32	0.89	0.66; 1.21	1.07	0.89; 1.28	1.27	1.06; 1.51
Q3	1.12	0.95; 1.33	1.29	1.13; 1.47	1.47	0.90; 2.40	1.12	0.97; 1.30	1.65	1.29; 2.10
Q4	1.10	0.90; 1.36	1.11	0.97; 1.27	1.27	0.88; 1.82	1.15	0.99; 1.35	1.29	1.03; 1.62
Q5 (most deprived)	1.18	0.97; 1.44	1.08	0.94; 1.25	1.15	0.75; 1.76	1.18	1.00; 1.39	0.98	0.77; 1.24
Spatial filter	1.38	1.23; 1.56	1.49	1.38; 1.62	2.59	0.69; 9.79	1.67	1.50; 1.85	1.03	0.99; 1.06
Pseudo R^2^	0.073		0.039		0.035		0.068		0.072	
Circulatory deaths										
PM_2.5_	1.01	0.96; 1.06	0.97	0.94; 0.99	1.15	0.99; 1.33	0.96	0.89; 1.03	1.06	1.01; 1.12
% adults aged 60 or more years	1.32	1.11; 1.57	1.46	1.30; 1.64	1.38	1.02; 1.86	1.27	1.11; 1.45	1.82	1.50; 2.19
% male	0.43	0.27; 0.67	1.08	0.95; 1.22	1.34	0.61; 2.95	0.70	0.55; 0.90	2.47	1.30; 4.68
MPI (quintiles)										
Q1 (least deprived)	Reference		Reference		Reference		Reference		Reference	
Q2	0.99	0.82; 1.20	1.12	1.02; 1.32	0.83	0.63; 1.09	1.05	0.87; 1.25	1.27	1.07; 1.49
Q3	1.07	0.90; 1.27	1.17	1.03; 1.32	1.27	0.81; 1.99	1.11	0.95; 1.31	1.59	1.27; 2.00
Q4	1.18	0.94; 1.47	1.05	0.93; 1.19	1.02	0.73; 1.44	1.20	1.00; 1.44	1.28	1.04; 1.57
Q5 (most deprived)	1.24	0.99; 1.55	1.00	0.88; 1.13	0.85	0.56; 1.28	1.13	0.94; 1.35	1.02	0.81; 1.28
Spatial filter	1.63	1.39; 1.91	1.73	1.60; 1.88	0.97	0.93; 0.99	1.60	1.42; 1.81	1.16	1.08; 1.24
Pseudo R^2^	0.069		0.070		0.038		0.054		0.090	
Respiratory deaths										
PM_2.5_	1.03	0.98; 1.08	0.99	0.96; 1.03	1.18	1.03; 1.34	0.99	0.93; 1.05	1.07	1.02; 1.13
% adults aged 60 or more years	1.38	1.18; 1.61	1.34	1.19; 1.49	1.36	1.02; 1.80	1.35	1.19; 1.52	1.86	1.54; 2.23
% male	0.42	0.29; 0.60	0.95	0.84; 1.06	0.84	0.52; 1.35	0.87	0.64; 1.19	1.53	0.96; 2.43
MPI (quintiles)										
Q1 (least deprived)	Reference		Reference		Reference		Reference		Reference	
Q2	1.09	0.89; 1.33	1.12	1.01; 1.26	1.02	0.75; 1.38	1.03	0.86; 1.22	1.21	1.02; 1.43
Q3	1.12	0.93; 1.35	1.21	1.07; 1.37	1.60	0.97; 2.64	1.12	0.97; 1.29	1.45	1.17; 1.80
Q4	0.97	0.78; 1.20	1.20	1.06; 1.36	1.28	0.91; 1.80	1.16	0.99; 1.35	1.20	0.96; 1.51
Q5 (most deprived)	1.17	0.96; 1.43	1.17	1.03; 1.33	1.15	0.78; 1.68	1.22	1.03; 1.44	0.93	0.74; 1.18
Spatial filter	1.65	1.40; 1.93	2.02	1.85; 2.21	2.59	0.92; 7.25	1.68	1.50; 1.87	1.19	1.09; 1.31
Pseudo R^2^	0.096		0.072		0.043		0.079		0.084	

95%CI: 95% confidence interval; IRR: incidence rate ratios; MPI: Colombian multidimensional poverty index.

**Figure 2:**
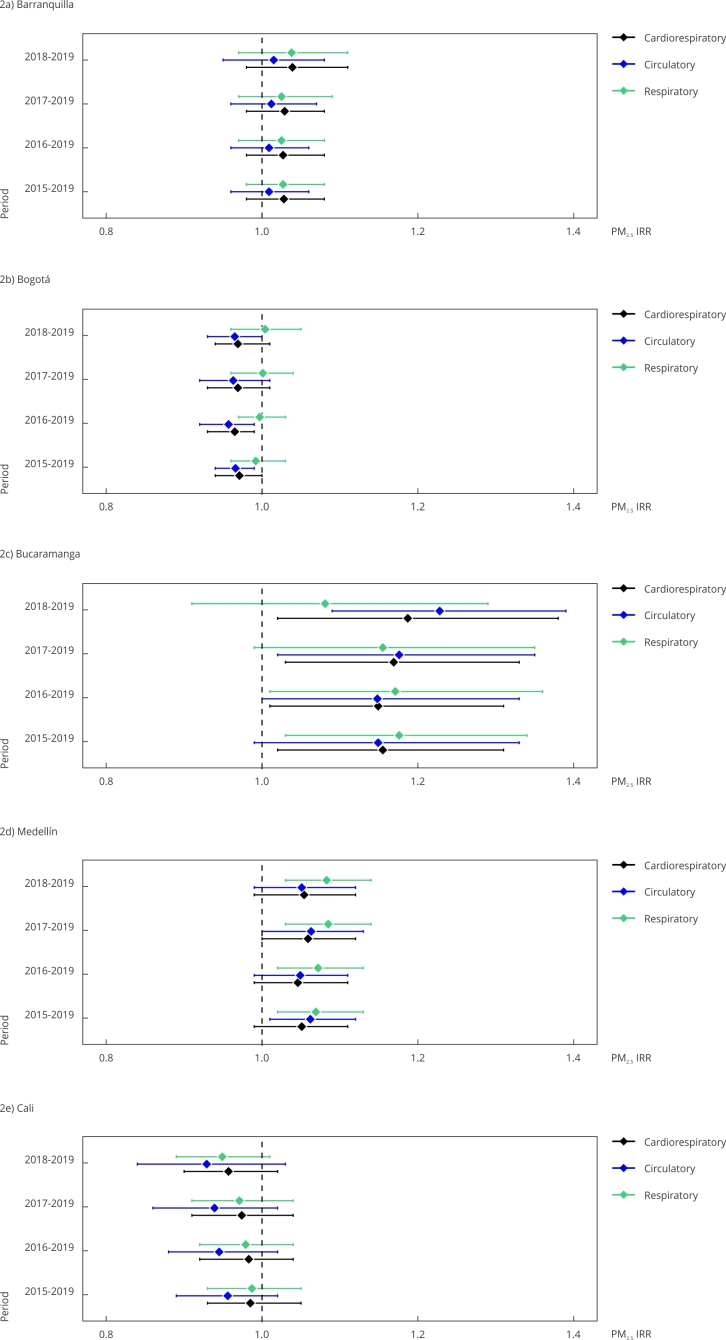
Sensitivity analysis: association between PM_2.5_ and cardiorespiratory, circulatory, and respiratory Bayesian mortality rates for five cities in Colombia considering different periods of registry (2015-2019, 2016-2019, 2017-2019, and 2018-2019).

Sensitivity analysis showed that the association between PM_2.5_ and cardiorespiratory mortality was consistent regardless of the window of time considered to estimate BMRs for all cities (Figure 2). Finally, the strength of the effect of PM_2.5_ on circulatory mortality became stronger in Bucaramanga as the window of time became closer to the year of exposure assessment, whereas we observed the opposite in Medellín.

IRR: incidence rate ratios. Note: all deaths are cardiorespiratory deaths.

## Discussion

Our findings show substantial variability in estimated long-term exposure to PM_2.5_ and cardiorespiratory mortality across CSs within Colombia’s five most populated cities between 2015 and 2019. Our analyses revealed statistically significant associations between exposure to ambient PM_2.5_ and cardiorespiratory mortality in Bucaramanga and Medellín, but not for Bogotá, Cali, and Barranquilla. To our knowledge, this is the first study in the Latin American and Caribbean region to analyze the association between long-term exposure to PM_2.5_ and mortality at the intraurban level.

Several explanations may account for why the correlations between PM_2.5_ exposure and cardiorespiratory mortality did not reach statistical significance in certain cities where previous short-term effect studies have found associations with morbidity and mortality ^14^ ^,^ ^30^ ^,^ ^31^. First, the PM_2.5_ exposure estimations obtained by the LUR models showed a higher explanatory power for Medellín and Bucaramanga than other cities, resulting in more accurate exposure assessments ^22^. This improved precision in exposure estimates may have strengthened the observed associations in these cities, rendering them statistically significant. Additionally, beyond the total PM_2.5_ concentrations, the specific PM_2.5_ toxicity in Medellín and Bucaramanga might have played a significant role in shaping the health outcomes. A PM_2.5_ oxidative potential analysis conducted in 2021 in the five cities revealed a higher burden of depletion of antioxidants such as ascorbate and glutathione ^32^, which indicates a potential higher toxicity of PM_2.5_ mixtures in Medellín and Bucaramanga. The biological effects of this heightened oxidative potential and their effects on morbidity and mortality warrant further investigation, as it may have critical implications for intraurban surveillance and understanding the differential health impacts of PM_2.5_ exposure across urban areas.

Results of the multivariable models uncover an essential ecological effect of age (% population 60 years old or more) in all cities and gender (% male), predominantly in Bogotá and Medellín, on cardiopulmonary mortality rates across CS, which aligns with expected patterns. Influence of age and gender on mortality is well-documented in the literature ^33^ ^,^ ^34^ ^,^ ^35^, and our findings further corroborate their significance in shaping health outcomes at the local level. Moreover, our study identified distinct disparities in cardiopulmonary mortality rates associated with MPI across the five cities. These observed inequalities in mortality rates have been previously documented in Colombia ^22^ ^,^ ^36^ ^,^ ^37^ ^,^ ^38^, indicating that the multidimensional poverty index is a robust measure in capturing important socioeconomic factors impacting health. Identifying such disparities underscores the need for targeted public health interventions aimed at reducing health inequities and improving the well-being of vulnerable populations in these urban areas.

Taken together, these results highlight the complex correlations between PM_2.5_ exposure and health outcomes at small-area levels in urban settings, suggesting the existence of diverse contributing factors and potential effect modifiers that warrant further investigation. The significant associations identified in Bucaramanga and Medellín emphasizes the importance of city-specific interventions and policies to mitigate the adverse health effects of long-term exposure to PM_2.5_ in these regions. Additionally, the observed high variability in PM_2.5_ exposure and cardiopulmonary mortality across CSs underlines the need for precise and localized exposure assessments when evaluating health risks associated with air pollution.

Research in air pollution epidemiology has undergone rapid advancement over the past decades, particularly in Europe, North America, and Asia ^8^ ^,^ ^9^. Large cohort studies have been conducted in these regions and documented the harmful effects of long-term exposure to air pollution on mortality, even at levels below World Health Organization (WHO) guidelines ^6^ ^,^ ^7^ ^,^ ^39^ ^,^ ^40^. However, Latin America has lagged behind in this domain, with significant knowledge gaps and limited progress. The scarcity of cohort and experimental studies and the lack of exposure assessments on smaller scales are notable shortcomings. Additionally, there is a dearth of research focused on analyzing health effects attributed to specific sources of pollutants like wildfires and traffic, which require nuanced investigations. Moreover, utilizing cutting-edge analytical tools and big data methodologies remains limited in this context ^16^. In Colombia, air pollution epidemiology has predominantly revolved around ecological time series studies, often conducted at the municipal level, seeking to identify health impacts associated with short-term exposure to air pollutants ^14^ ^,^ ^30^ ^,^ ^41^. While these studies have contributed valuable insights, it is crucial to broaden the scope of research efforts and adopt more comprehensive approaches to better understand the multifaceted implications of air pollution on public health in Colombia and Latin America.

This study has several limitations that should be acknowledged. First, the ecological nature of the design, being a cross-sectional study rather than a cohort study, restricts the establishment of causal inferences for the associations observed. While exposure and outcome were measured at the small-area level, the lack of individual-level follow-up data on exposure over time hinders a more comprehensive understanding of temporal relations. Second, the temporal relation between exposure and outcome raises potential concerns. Use of the LUR 2021 model to estimate long-term exposure from 2015 to 2019 assumes stability in PM_2.5_ levels during the study period. While this assumption aligns with the data from air quality reports for the last years ^42^, there may be temporal changes in air pollution levels that this approach cannot fully capture. Despite conducting sensitivity analysis over different periods, no substantial association changes were identified for the entire study duration, but the potential for subtle temporal fluctuations remains a limitation.

Another limitation is that being a cross-sectional ecological study, our investigation did not account for population mobility and its potential impact on exposure changes. Data from a recent cohort of children in Medellín and Bogotá reported that most families remain in the same residence from pregnancy to 5 years of age (90% and 66%, respectively), with a significant portion involving intra-city mobility (60% and 86%, respectively) ^43^. In our study, unless population mobility had occurred following a systematic pattern, we should expect an underestimation of our association estimates. Further, we could not assign an exposure estimate to all fatal cases due to missing or incomplete address information; however, the amount of not georeferenced outcomes was low (< 15%), and unless it showed a systematic pattern across exposure levels, the introduction of bias to the association estimates is unlikely. Lastly, and also related to our study design, residual confounding cannot be fully ruled out due to the adjustment of regression models for group-level covariates (i.e., % population 60 years old or more, % male, etc.).

This study also has several notable strengths that enhance the validity and reliability of our findings. One key strength is the use of small-area level exposure and outcome data, allowing for a more localized analysis than traditional municipality-level studies. By examining PM_2.5_ exposure and cardiopulmonary mortality at the small-area level, we were able to capture variations and patterns that might have been masked in broader geographical analyses. Additionally, the PM_2.5_ LUR model showed low variability within small areas, ensuring a more homogeneous exposure assessment which contributes to the accuracy of our results. Another strength lies in the specific health outcomes analyzed, which are well-established and consistently associated with air pollution effects. This robust selection of cardiorespiratory mortality as the primary health outcome increases the relevance and reliability of our findings, as it aligns with the existing body of literature on the impact of air pollution on human health. Moreover, we included and carefully controlled for the main ecological confounders of mortality such as population structure and socioeconomic conditions. By accounting for these crucial factors, we mitigated potential sources of bias and ensured that our observed associations between PM_2.5_ exposure and cardiorespiratory mortality were more likely to be genuine and not confounding artifacts.

## Conclusions

Our study provided valuable insights into the intricate interplay between PM_2.5_ exposure and cardiorespiratory mortality in urban environments, shedding light on potential vulnerability hotspots and guiding targeted public health interventions in Colombia. Moving forward, our findings emphasize the need for targeted public health interventions tailored to specific cities and the importance of incorporating localized exposure assessments to better understand the implications of air pollution on cardiopulmonary health in urban environments. Continued research efforts in air pollution epidemiology in Latin America focused on prospective cohort studies and advanced analytical tools are crucial to address existing knowledge gaps and inform evidence-based policies for improved public health outcomes.
