# Supplementary material for: Long-term exposure to PM2.5 and cardiorespiratory mortality: an ecological small-area study in five cities in Colombia
Source: Cad Saude Publica. 2025 Apr 25;41(4):e00071024. doi: 10.1590/0102-311XEN071024 (PMC12037112; doi:10.1590/0102-311XEN071024)

SUPPLEMENTARY MATERIAL

Figure S1. Bayesian circulatory mortality rates by city in Colombia, 2015-2019.

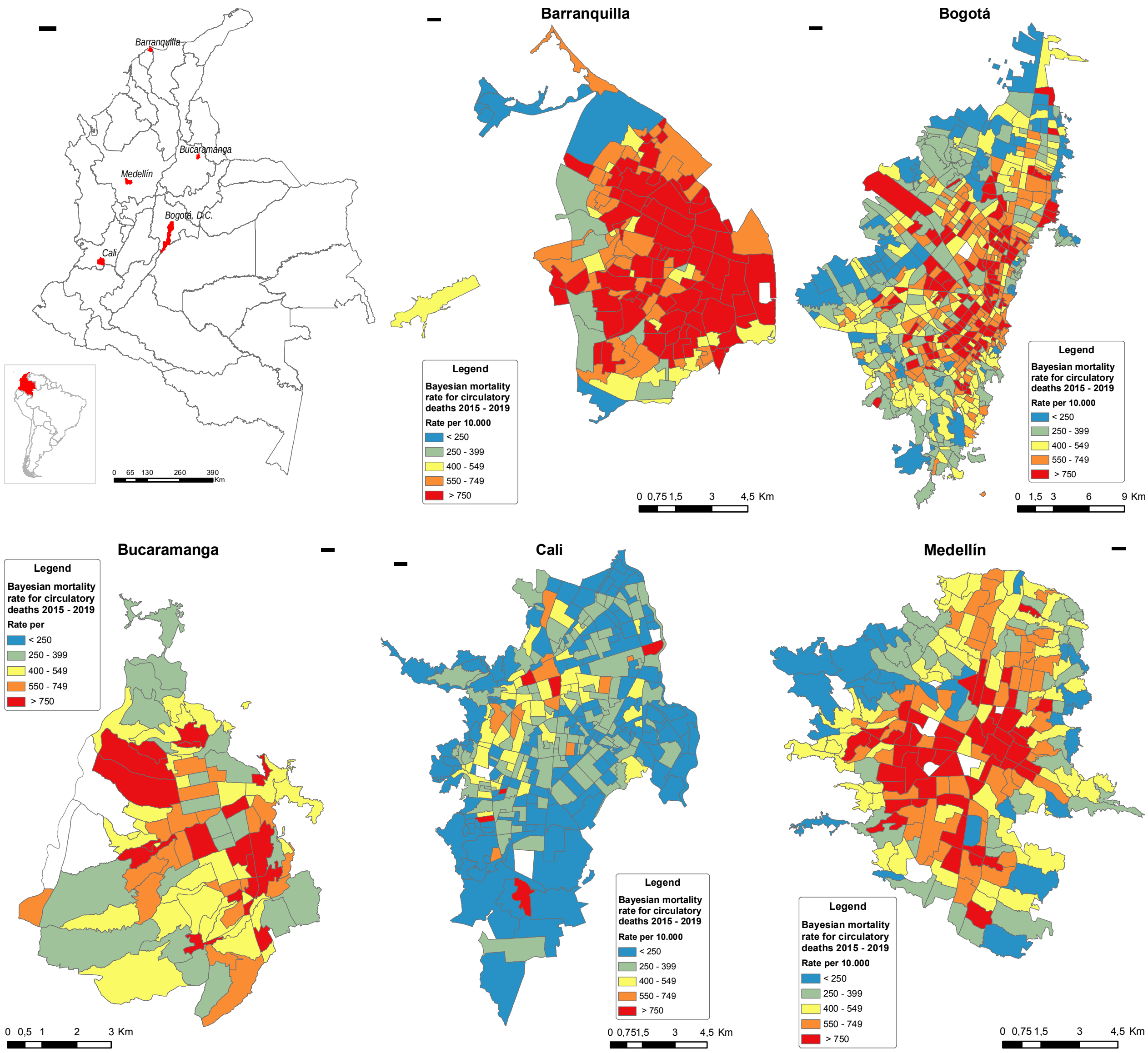

Figure S2. Bayesian respiratory mortality rates by city in Colombia, 2015-2019.

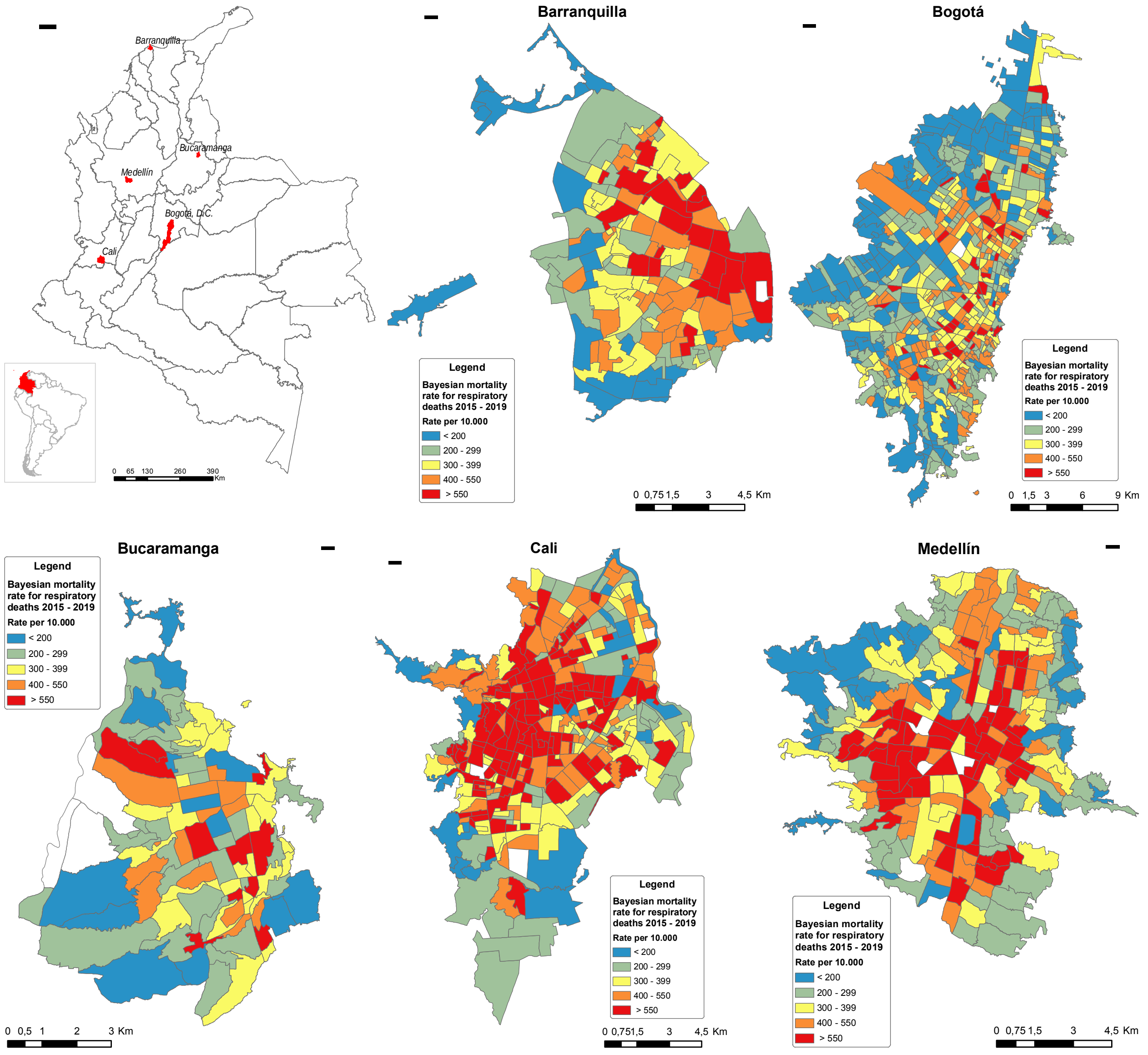

Supplement: Supplementary file 1 [file 1678-4464-csp-41-04-EN071024-s.pdf]
